# Multimodal semi-supervised learning for online recognition of multi-granularity surgical workflows

**DOI:** 10.1007/s11548-024-03101-6

**Published:** 2024-04-01

**Authors:** Yutaro Yamada, Jacinto Colan, Ana Davila, Yasuhisa Hasegawa

**Affiliations:** 1 Department of Micro-Nano Mechanical Science and Engineering, Nagoya University, Furo-cho, Chikusa-ku, Nagoya, Aichi 464-8603 Japan; 2 Institutes of Innovation for Future Society, Nagoya University, Furo-cho, Chikusa-ku, Nagoya, Aichi 464-8601 Japan

**Keywords:** Semi-supervised learning, Multimodal learning, Surgical workflow recognition, Robotic surgery

## Abstract

**Purpose** Surgical workflow recognition is a challenging task that requires understanding multiple aspects of surgery, such as gestures, phases, and steps. However, most existing methods focus on single-task or single-modal models and rely on costly annotations for training. To address these limitations, we propose a novel semi-supervised learning approach that leverages multimodal data and self-supervision to create meaningful representations for various surgical tasks. **Methods** Our representation learning approach conducts two processes. In the first stage, time contrastive learning is used to learn spatiotemporal visual features from video data, without any labels. In the second stage, multimodal VAE fuses the visual features with kinematic data to obtain a shared representation, which is fed into recurrent neural networks for online recognition. **Results** Our method is evaluated on two datasets: JIGSAWS and MISAW. We confirmed that it achieved comparable or better performance in multi-granularity workflow recognition compared to fully supervised models specialized for each task. On the JIGSAWS Suturing dataset, we achieve a gesture recognition accuracy of 83.3%. In addition, our model is more efficient in annotation usage, as it can maintain high performance with only half of the labels. On the MISAW dataset, we achieve 84.0% AD-Accuracy in phase recognition and 56.8% AD-Accuracy in step recognition. **Conclusion** Our multimodal representation exhibits versatility across various surgical tasks and enhances annotation efficiency. This work has significant implications for real-time decision-making systems within the operating room.

**Supplementary Information:**

The online version contains supplementary material available at 10.1007/s11548-024-03101-6.

## Introduction

In robot-assisted minimally invasive surgery (RMIS), intraoperative context-aware assistance has gained significant attention beyond current passive augmentation, such as precise tool control and superior visualization. This support can enable intelligent scheduling and resource management [1], surgical training platforms [2], and autonomous robotic assistance [3]. For optimal online surgical assistance, it is essential to understand the surgical workflow and surgeons’ actions and intentions at different levels of granularity, such as states, procedures, phases, steps, activities, gestures, and dexemes [4]. However, this is a challenging task due to the complexity of the surgical workflow, the variability among surgeons, and the diversity of multimodal data sources.

Most existing methods for surgical recognition rely on supervised learning, which requires costly annotations for training. Moreover, they are usually limited to single-modality and/or single-task models, which cannot capture the holistic aspects of surgery. In contrast, representation learning aims to learn meaningful and general representations for multiple tasks by effectively capturing structures and relationships underlying the data without the need of annotations [5]. In robotic surgery, visual data provides a comprehensive view of the surgical procedure, while kinematic data captures the precise movements and manipulations of the tools and the surgeon. These modalities contain unique and complementary information that can enhance surgical scene understanding. Furthermore, the surgical process is sequential and multimodal, as the surgeon performs multi-stage tasks while interacting with different modalities. This structure is suitable for representation learning that leverages both temporal and multimodal information.

In this work, we propose a semi-supervised learning method that combines representation learning with supervised classifiers for holistic surgical scene understanding. Our representation learning method employs time contrastive learning to extract spatiotemporal representations from visual data without any labels, which are then integrated into a shared representation by the multimodal variational autoencoder (MVAE) considering the complementary relationships between modalities. This results in a shared representation that encodes various facets of surgery and demonstrates its versatility across multi-granularity workflow.

Designed for online inference, multimodal integration, and multi-granularity recognition, our model can provide a real-time comprehensive understanding of robotic surgery in contrast to traditional offline models. This capability enables the integration of extensive online context-aware support, ranging from time and resource management based on a high-level understanding to autonomous robotic assistance that are adapted to fine-grained gestures. Moreover, our semi-supervised method effectively addresses the challenge of limited annotations in surgical applications.

## Related work

### Surgical workflow recognition

The evolution of surgical workflow recognition began with supervised graphical models [6], transitioning to deep learning with unimodal approaches like sequential models [7] and convolutional neural networks [8]. While foundational, these models were constrained by their focus on unimodal data. On the other hand, multimodal models, such as Fusion-KV [9] with weighted voting, MRG-Net [10] utilizing a relational graph network, and MA-TCN [11] applying multimodal attention, have achieved high accuracy in gesture recognition. However, their reliance on labeled data limits their applicability.

In contrast, semi-supervised models can extract valuable insights from large unlabeled data or efficiently use them alongside limited annotations. SurgSSL [12] achieved on-par performance with fully supervised models, using only 50% labeled data for surgical workflow recognition, underscoring the importance of capturing sequential patterns. Tanwani et al. [13] utilize contrastive learning, while Wu et al. [14] employ cross-modal prediction of kinematic data from optical flow, to extract meaningful representations. However, these semi-supervised methods rely solely on visual data. Even the cross-modal method presented in [14] utilizes multimodal data exclusively during training and operates as a unimodal model during inference.

Our model stands out from previous works by incorporating both kinematic and visual data in a semi-supervised setting. This approach overcomes the limitations of existing methods that heavily depend on single inputs or fully labeled data.

### Contrastive learning for video understanding

Video understanding without supervision is a challenging task that requires a framework that can capture both the static content and the dynamic context of the images. Contrastive learning is a technique that learns to bring similar samples closer and push dissimilar ones apart in a latent space. It has achieved remarkable progress in self-supervised image recognition. Recent research has extended this approach to learning spatiotemporal features in video data.

For instance, SeCo [15] learns multiple aspects of video through inter-frame/intra-frame discrimination and temporal order validation. TCLR [16] leverages two loss functions for discriminating between non-overlapping clips from the same video and between timesteps within the clip’s feature map to obtain local and global representation. Notably, both SeCo and TCLR have showcased remarkable performance in action recognition, a global-level understanding task. Time-contrastive networks (TCN) [17] learn to find commonalities in temporal neighbors and differences in temporally distant points using multiple or single viewpoints. TCN has enabled reinforcement learning for robot’s human imitation, demonstrating its ability to capture the sequential flow and detailed motion of the video.

**Fig. 1:**
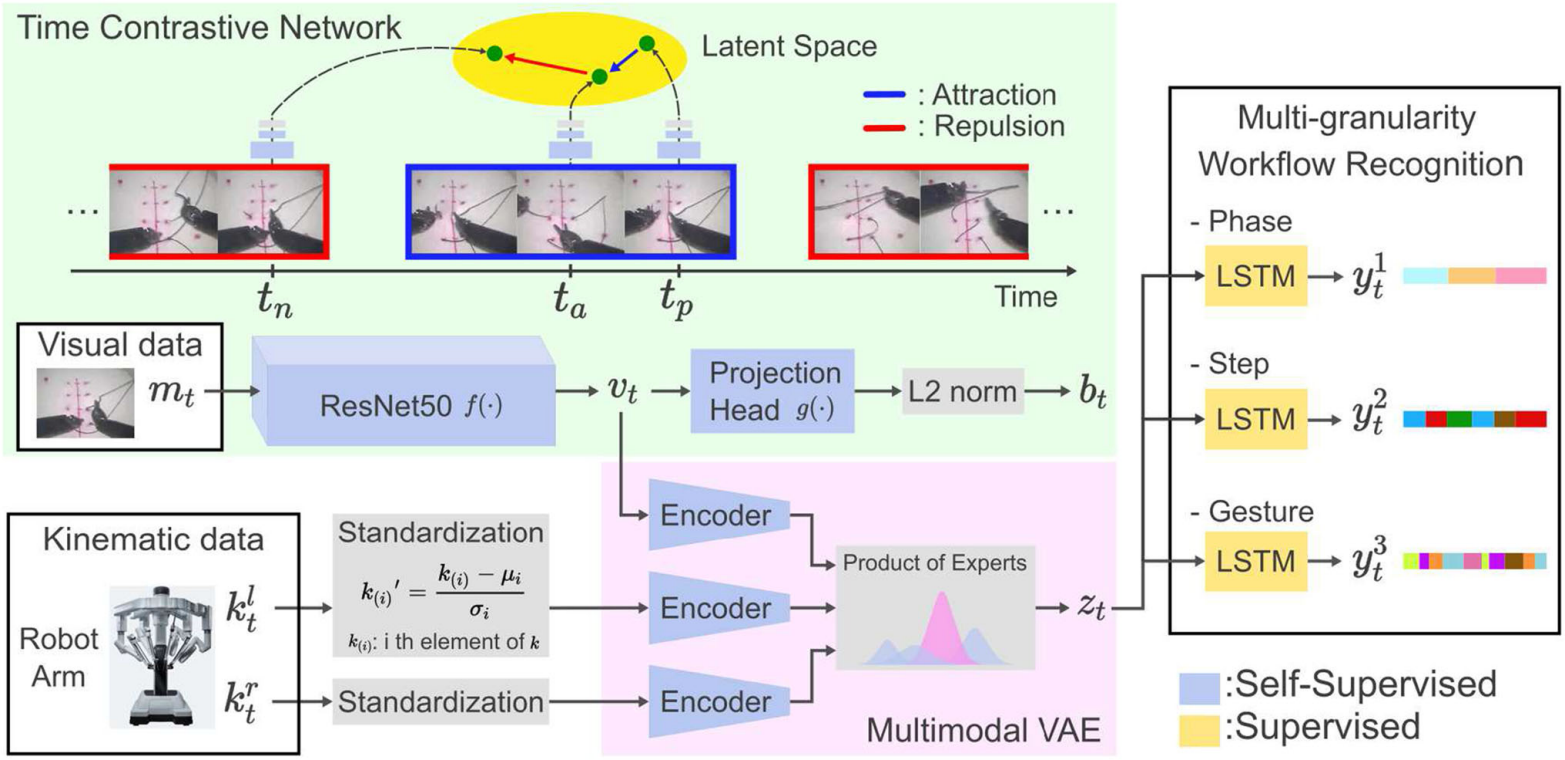
Overview of the proposed semi-supervised model for multi-granularity workflow recognition

## Method

We consider a multimodal dataset $X = \{\varvec{m}_{d,t},\varvec{k}^l_{d,t}, \varvec{k}^r_{d,t}\}_{d=1,t=1}^{D, T_d}$, containing *D* demonstrations with a duration of $T_d$. This dataset comprises visual data $\varvec{m}_{d,t}$ and kinematic data $\varvec{k}_{d,t}$ from the right and left robot arms. For simplicity, subscripts *d* and *t* may be omitted in the rest of the paper. Our model consists of two components: self-supervised representation learning and supervised learning (Fig. 1). First, the spatiotemporal feature extractor generates visual features $\varvec{v}$ from the video frame $\varvec{m}$. Then, MVAE obtains the shared representation $\varvec{z}$ by combining $\varvec{v}$ with kinematic data $\{\varvec{k}^l, \varvec{k}^r\}$. Finally, $\varvec{z}$ is fed into supervised LSTMs to recognize multi-granularity workflow.

### Spatiotemporal feature extractor

We introduce a single-view version of TCN [17] into the network architecture inspired by [18] to obtain spatiotemporal properties in robotic surgery. The process starts with the base encoder $f(\cdot )$, a ResNet50 without the fully connected layer, which transforms input images $\varvec{m}$ into visual features $\varvec{v}$. Next, $\varvec{v}$ are further processed by an MLP projection head $g(\cdot )$ and subjected to L2 normalization, resulting in features $\varvec{b}$. This enables us to preserve more information in visual features $\varvec{v}$ than $\varvec{b}$ [18].

The objective of TCN is to ensure that a feature vector $\varvec{b}_{d,t_a}$ (anchor) is closer in the latent space to its temporal neighbor $\varvec{b}_{d,t_p}$ (positive) within a positive range $r_p$, than a temporally distant point $\varvec{b}_{d,t_n}$ beyond a margin range $r_m$, using a triplet loss [19].

1 $$\begin{aligned} \mathcal {L}_{\text {triplet}} = \left[ ||\varvec{b}_{d,t_a} - \varvec{b}_{d,t_p}||_2^2 - ||\varvec{b}_{d,t_a} - \varvec{b}_{d,t_n}||_2^2 + \alpha \right] _+ \end{aligned}$$

Here, $\alpha$ is a margin that is enforced between positive and negative pairs, and the loss is averaged over all triplets. For optimization, TCN needs to recognize the similarities between temporal neighbors and dissimilarities between distant points. This encourages the model to focus on temporal variant factors while ignoring static background. In addition, temporal neighbors are situated closer in the latent space than distant points, facilitating sequential understanding. TCN captures the situation and progression of manipulated objects, such as the robot and the processed area.

### Multimodal variational autoencoder

We utilize MVAE to project data from *M* modalities into a shared latent space. These modalities contain the visual feature and kinematic data in robotic surgery, denoted as $X=\{\varvec{x}_m\}_{m=1}^M=\{\varvec{v},\varvec{k^l},\varvec{k}^r\}$, where *M* equals three. MVAE extends the ability of standard VAEs to handle multimodal data sources. It assumes each modality is conditionally independent given $\varvec{z}$. This assumption reflects the complementary relationships between modalities, with each modality capturing a different aspect of the comprehensive surgical situation represented by $\varvec{z}$.

In this study, we obtain individual $\varvec{z}$ from each unimodal encoder and aggregate them into a shared representation $\varvec{z}$ by a product of experts (PoE) [20, 21]. MVAE is trained by minimizing the loss function that combines the standard VAE loss with weight $\beta$ [22] and its extension loss for multimodal VAE with weights $\lambda _m$ and $\beta$ [20], preventing specific modalities from dominating the shared representation $\varvec{z}$.

2 $$\begin{aligned}{} & {} \mathcal {L}_{MVAE}(X) = \sum _{\varvec{x}_m \in X} - \mathbb {E}_{q_\phi (\varvec{z}|\varvec{x}_m)} [\log p_\Theta (\varvec{x}_m|\varvec{z})]\nonumber \\{} & {} \quad + \beta D_{KL}(q_\phi (\varvec{z}|\varvec{x}_m) || p(\varvec{z}))\nonumber \\{} & {} \quad - \mathbb {E}_{q_\phi (\varvec{z}|X)} [\sum _{\varvec{x}_m\in X}\lambda _m \log p_\Theta (\varvec{x}_m|\varvec{z})]\nonumber \\{} & {} \quad + \beta D_{KL}(q_\phi (\varvec{z}|X) || p(\varvec{z})) \end{aligned}$$

### LSTM classifier

We train LSTMs independently for workflow recognition at each of the *G* granularity levels, with workflow labels $\varvec{y}_{d,t}^g$ corresponding to each level. LSTMs capture the sequential nature of the surgical process from $\varvec{z}$ and predict the corresponding labels. They retain relevant information, update it with new data, and preserve both short-term and long-term context, making them suitable for sequential recognition. LSTMs are trained using cross-entropy losses as discriminative models for online workflow recognition at each granularity, denoted as $p(\varvec{y}^g_{d,t}|\varvec{z}_{d,1:t})$.

## Experimental setup

We evaluate the proposed model’s performances in multi-granularity workflow recognition through *gesture recognition* on JHU-ISI Gesture and Skill Assessment Working Set (JIGSAWS) dataset [23], and *phase recognition* and *step recognition* on MIcro-Surgical Anastomose Workflow recognition on training sessions (MISAW) dataset [24].

### Datasets and evaluation metrics

For gesture recognition, we use Suturing (SU), Knot Tying (KT), and Needle Passing (NP) tasks from the JIGSAWS dataset, which were recorded using the da Vinci surgical system. Eight surgeons, categorized into three skill levels based on robotic surgical experience, performed each task five times. The dataset provides synchronized data at 30 Hz, including kinematic data from two robot arms (Patient-Side Manipulators: PSMs) and two controllers, as well as stereo video. Each frame is labeled using 15 common vocabularies across all tasks. For model training, we used the right camera frames and normalized robot arms data to have zero mean and unit variance.

The dataset includes two cross-validation strategies: *leave-one-supertrial-out* (LOSO) and *leave-one-user-out* (LOUO). LOSO reserves one trial from each user for testing, while LOUO utilizes one user’s trials for testing. We evaluated our gesture recognition performance using both cross-validation methods by frame-wise accuracy (Acc) and edit score (Edit*). The edit score, computed as the Levenshtein distance between the true and predicted segments and normalized to [0,100], assesses segment order rather than timing, penalizing misordering and over-segmentation [4].

The MISAW dataset, designed for surgical workflow recognition, includes synchronized stereo video and kinematic data from two robot arms at 30Hz. It offers annotations for 27 demonstrations across three granularities: phase, step, and activity. We evaluated our model at the phase (3 classes) and step (7 classes) levels, using balanced application-dependent accuracy (AD-Accuracy) as in the original paper, which assigns equal importance to each class and allows for a transition delay of $d=500 ms$.

### Implementation details

All modules were implemented using PyTorch and trained independently on an NVIDIA RTX A6000 GPU, with ReLU activation and the AdamW optimizer. The same hyperparameters were applied to all experiments.

*TCN:* It was trained at 3 FPS, with a positive range of 6, a margin range of 12, a batch size of 128, $\alpha$ of 0.2, and an embedding layer with 32 dimensions for the projection head, following [13]. We used a ResNet50 model pre-trained on ImageNet and added a projection head with hidden layers {1000, 500} instead of the fully connected layer. It was trained for 100 epochs with a learning rate of 0.0005 and a weight decay of 0.01. Features were saved at 30 FPS after training.

*MVAE:* It includes dense layers for each robot arm (hidden layers: {200, 500}) and visual features (hidden layer: {1000}), and a shared representation vector with dimension 500. It was trained with $\beta$ of 0.1, a learning rate of 0.0001, and a batch size of 256 at 30 FPS. Training was stopped when the validation loss did not decrease for fifteen epochs between 25 and 300 epochs.

*LSTM:* It has a single layer with 300 hidden units, applying 50% dropout to input and output layers. It was optimized with a learning rate of 0.001, a weight decay of 0.05, and a batch size of 3 on 70 epochs at 5 FPS and 30 FPS.

## Results and discussion

### Visualization of latent representations

**Fig. 2:**
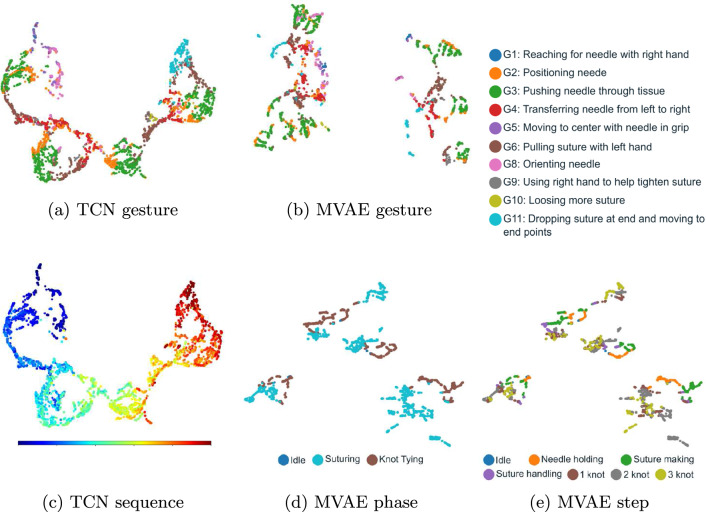
UMAP visualization of $\varvec{v}$ and $\varvec{z}$: **a** and **b** gesture labels obtained from TCN and MVAE respectively; **c** Normalized frame indexes on JIGSAWS SU; **d** and **e** phase and step labels on MISAW

To evaluate the abilities of TCN and MVAE, their representations were projected to 2D using UMAP [25] and visualized in normalized frame indexes to observe the sequence and workflow labels, as shown in Fig. 2. The visual feature $\varvec{v}$ in Fig. 2a and c illustrates TCN’s ability to capture both the video sequence and gestures, respectively, implying that our model can effectively break down the surgical process into gesture-level components. Figure 2b, d and e shows that MVAE creates solidified clusters for gesture, phase, and step. These results suggest that the proposed representation learning can capture the hierarchical workflow without any labels by effectively integrating and interpreting multimodal data.

**Table 1:** Comparison of gesture recognition performance with online applicable models on JIGSAWS (mean and standard deviation, %)

Model	Condition	SU		KT		NP	
	Type/Valid/Mod	Acc	Edit*	Acc	Edit*	Acc	Edit*
SC-CRF [6]	Sup/LOUO/Kin	81.7	–	79.0	–	**74**.**8**	–
Forward LSTM [7]	Sup/LOUO/Kin	80.5(6.2)	75.3	–	–	–	–
3D-CNN [8]^1^	Sup/LOUO/Vis	81.8	58.7	–	–	–	–
Fusion-KV [9]	Sup/LOUO/	86.3	87.2	–	–	–	–
	Kin, Vis						
MRG-Net [10]	Sup/LOUO/	**87**.**9**(4.2)	**89**.**3**(5.2)	**88**.**1**(3.8)	**87**.**0**(6.8)	–	–
	Kin, Vis						
MA-TCN [11]	Sup/LOUO/	83.4(5.8)	81.6(7.6)	–	–	–	–
(casual)	Kin, Vis						
Ours (top: 30 FPS	Semi/LOUO/	83.3(8.3)	61.8(17.3)	75.5(14.6)	51.7(18.2)	64.3(14.0)	37.2(12.7)
bottom: 5 FPS)	Kin, Vis	82.4(9.2)	76.7(15.2)	77.8(12.8)	63.9(20.9)	63.0(13.2)	**59**.**4**(13.2)
Motion2vec [13]^2^	Semi/LOSO/Vis	84.4	–	–	–	–	–
Cross-modal [14]^3^	Semi/LOTO/	68(3)	–	64(3)	–	64(3)	–
	(Kin), Vis						
Ours (top: 30 FPS	Semi/LOSO/	**87**.**9**(6.6)	71.5(15.9)	77.2(13.8)	53.8(20.3)	71.5(17.3)	47.7(19.4)
bottom: 5 FPS)	Kin, Vis	87.3(6.9)	**82**.**9**(14.1)	**78**.**4**(15.4)	**67**.**2**(23.0)	**71**.**8**(17.3)	**65**.**7**(17.3)

The bold values indicate the highest performance among the models that use the same cross-validation strategy within each task (SU, KT, or NP)

‘Sup’: Supervised learning, ‘Semi’: Semi-supervised learning, ‘Kin’: Kinematic data, ‘Vis’: Visual data,

^1^For the online condition, the model without looking ahead was selected

^2^Result of a KNN classifier for the online condition and average of 4 iterations on the LOSO test set

^3^Segmental classification on leave-one-trial-out cross-validation, Kin is only used for training

### Gesture recognition

#### Comparison with other online applicable models

We compared the performance of our semi-supervised model in gesture recognition with state-of-the-art models that can be applied to online scenarios, where future data are not available. To ensure a fair comparison, we used LOUO cross-validation for supervised models and LOSO cross-validation for semi-supervised models (Table 1).

Compared with the supervised models, our model outperforms the unimodal models in accuracy at 5 and 30 FPS on SU, even though it is semi-supervised. The effectiveness of a multimodal representation is visible in the accuracy improvement compared to a Forward LSTM [7], which only uses kinematic data. When compared with semi-supervised models, the proposed approach surpassed them in all tasks by approximately 3% or more, demonstrating its superiority within online semi-supervised approaches. Furthermore, multimodal models like ours generally outperformed unimodal ones, underscoring the advantages of multimodal integration, which considers the relationship between modalities.

Note that the data show a performance decrease on KT and NP, with accuracies dropping by 7.8% to 19.0% compared to SU, alongside greater variability. This mirrors the trend of lower KT and NP performance found in previous research [6], worsened by non-optimized hyperparameters and the representation’s alignment challenges in complex tasks. Similarly, segmental coherence represented by the edit score could be affected by several factors. A higher frame rate of recognition can increase the likelihood of introducing noise, disrupting segmental coherence. Edit score is affected by noise, as even one wrong prediction can become an additional segment and lower the score considerably. Only downsampling the FPS can provide large benefit, as shown in Table 1, where using 5 FPS (as in [7, 8, 11]) instead of 30 FPS improved edit scores. Offline inference, which is excluded in this comparison due to the use of the entire sequence, could also enhance prediction consistency and performance compared to online inference. Our task-agnostic representation, which encompasses information for various tasks, contributes to the instability in segmental prediction, leading to our high variance. Further analysis of the NP task and details of comparison targets are available in the supplementary material.

The proposed model also often fails to recognize the gestures G9 and G10 in SU and NP. The proposed representation learning may overlook them due to their under-representation: G9 and G10 appear in only 6.7% and 1.7% of SU frames, and 5.9% and 0.2% of NP frames, respectively. Training with a larger, unlabeled dataset or balanced classification loss may improve our model’s ability to identify such gestures.

**Table 2:** Gesture recognition performance of different input modalities on JIGSAW SU for LOUO cross-validation at 30 FPS

Modalities	Acc	Edit*
l-PSM, r-PSM	73.4	41.7
Visual	75.6	52.6
Visual, l-PSM	80.9	60.8
Visual, r-PSM	81.5	**66**.**1**
Visual, l-PSM, r-PSM	**83**.**3**	61.8

l-PSM: left-PSM, r-PSM: right-PSM

The bold values indicate the highest performance among different input modalities

#### Ablation analysis for multimodal integration

To evaluate the effect of multimodal integration, we performed five LOUO cross-validations on JIGSAWS SU with different input modalities for MVAE. Table 2 shows the performance for various modality combinations, indicating that MVAE effectively takes advantage of multimodal data. This observation is consistent with PoE’s property that aggregation of more modalities leads to a sharper posterior distribution [5]. Adding {l-PSM} to {Visual, r-PSM} improved accuracy but reduced the edit score. This suggests that while additional information enhances recognition of the current scene, it may introduce noise unrelated to workflow, disrupting the coherence of the segmented output. Strategies such as FPS reduction or post-processing based on prior knowledge can mitigate this issue and should be carefully tailored and applied to specific applications to ensure stable segmental consistency.

#### Effect of decreasing the amount of annotation

**Fig. 3:**
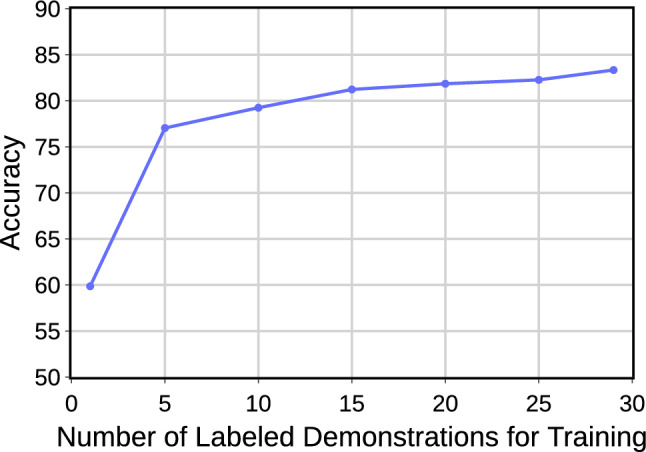
Effect of the amount of labeled data used for training on the JIGSAW SU for LOUO cross-validation at 30 FPS

To showcase the performance with limited annotations, we experimented using LOUO cross-validation, which involved fewer labeled demonstrations for training. We trained the representation learning components with the entire data (w/o annotation) and trained LSTM with varying amounts of annotation on JIGSAWS SU. Our findings show that with annotations from only 15 demonstrations, roughly half of the total training data, the model consistently maintained a high accuracy of 81.2% (Fig. 3). This accuracy is only 2.1% lower than the 83.3% achieved with the entire dataset and still comparable to the supervised models in Table 1. These results suggest a high annotation efficiency, which enables improved generalizability when large datasets with partial labeling are available, thereby reducing the need for costly annotations.

**Table 3:** Comparison of phase and step recognition performance on MISAW

Model (Team)	Networks	Mod	AD-Accuracy	
			Phase	Step
UniandesBCV	SlowFast,CNN	V	89.45	60.21
Wr0112358	CNN	V	91.60	63.74
MedAIR	MRG-NET [10]	K,V	**96**.**53**	**84**.**02**
IMPACT	CNN	K,V	80.66	46.48
Ours (30 FPS)	CNN,VAE,RNN	K,V	84.03	56.78

The bold values indicate the highest performance among models

‘K’: Kinematic data, ‘V’: Visual data Refer to the supplementary material and [24] for details

### Phase and step recognition

To assess higher granularity levels than gestures, we benchmarked our phase and step recognition against models presented in the MISAW report [24] using a hold-out method. These models, employing supervised learning techniques, include both uni-task and multi-task methods. Our comparison focused exclusively on uni-task models to eliminate multi-task learning effects, aiming for a direct performance assessment at individual granularity level (Table 3). Although our performance did not consistently surpass these supervised models, it was still competitive. This result shows that our task-agnostic representation can retain high-level workflow information without being limited to fine-grained levels, leading to a more holistic surgical understanding.

**Fig. 4:**
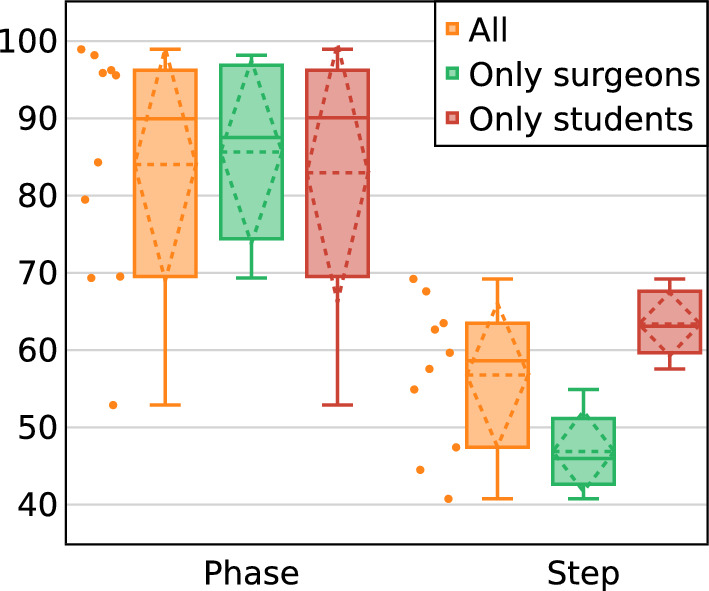
Test AD-Accuracy on MISAW: dash lines represent mean and sd

Figure 4 shows the AD-Accuracy obtained for the phase and step recognition tasks depending on participants’ skills. Phase recognition excelled in half the demonstrations (5 in total) with AD-Accuracy over 95%, yet the lowest score was around 50% with no notable difference between surgeons and students. On the other hand, in step recognition, there was a clear distinction: Student demonstrations average 63.4% AD-Accuracy, while surgeon demonstrations average 46.8%. These results highlight a shortfall in generalization for certain instances and skill levels.

Training data of the MISAW dataset comprises 7 demonstrations by surgeons and 10 by students, with students exhibiting longer sessions. Surgeons’ videos average 2.5 min in length, while students’ average 4.0 min. This relatively small dataset size, combined with great variability in skill and instance differences, likely contributes to the increased instability of the proposed model. Although self-supervised learning methods originally aim to enhance generalizability from large unlabeled data, high variability in a small dataset poses a significant challenge to find underlying general patterns from the data. In contrast, supervised learning methods can mitigate the effect of variability with explicit label guidance, resulting in a performance difference compared to our self-supervised learning.

## Conclusion and future work

In this study, we developed a multimodal self-supervised representation learning method capable of understanding surgical workflow across various granularity levels, from gestures to phases. The performance achieved across these tasks is comparable to fully supervised models designed for specific tasks, highlighting the versatility of our representation. This capability gives the model a broader perspective and allows for intelligent surgical platforms that provide extensive context-aware support, ranging from decision-making and resource management to autonomous robotic assistance and error detection.

Our approach holds promise in addressing surgical annotation challenges. It maintains high performance with partially labeled data and does not rely on labels for representation learning. This enables the use of large unlabeled data and continuous performance improvement. Another key benefit is the ability to handle multiple modalities, promoting enhanced expressiveness and adaptability. This capability can be extended to integrating new modalities, such as surgeon gaze, voice, and other interface information. It is also useful for handling information from multiple robot arms, which is invaluable in scenarios where a human operates two or more robots simultaneously, or when additional robots provide autonomous assistance.

Nevertheless, it is important to note that some tasks may still exhibit performance gaps between supervised models and the proposed semi-supervised model, especially in highly complex scenarios. The limited size of datasets like JIGSAWS and MISAW currently hinders a full exploration of self-supervised methods’ capabilities. Current robotic surgical setups allows for rich self-supervised signals, including sequential properties and synchronization of diverse multimodal data. Developing larger multimodal workflow datasets, expanded through partial labeling, will benefit self-supervised methods and enable the application of successful strategies from computer vision and natural language processing. Future research should explore transfer learning and fine-tuning schemes, to enhance generalizability and applicability. Additionally, our experiments were limited to benchmark tests. We also plan to collect real-world datasets and examine this model’s behavior and responsiveness in real time.

### Supplementary Information

Below is the link to the electronic supplementary material.

Supplementary file 1 (pdf 3015 KB)
