# Supplementary material for: Multimodal semi-supervised learning for online recognition of multi-granularity surgical workflows
Source: Int J Comput Assist Radiol Surg. 2024 Apr 1;19(6):1075–83. doi: 10.1007/s11548-024-03101-6 (PMC11178653; doi:10.1007/s11548-024-03101-6)
Supplement: Supplementary file 1 — (pdf 3015 KB) [file 11548_2024_3101_MOESM1_ESM.pdf]

Supplementary Material: Multimodal Semi-Supervised Learning for Online Recognition of Multi-granularity Surgical Workflows

International Journal of Computer Assisted Radiology and Surgery

Yutaro Yamada[1*], Jacinto Colan[1], Ana Davila[2], Yasuhisa Hasegawa[1]

[1*]Department of Micro-Nano Mechanical Science and Engineering, Nagoya University, Furo-cho, Chikusa-ku, Nagoya, 464-8603, Aichi, Japan.

[2]Institutes of Innovation for Future Society, Nagoya University, Furo-cho, Chikusa-ku, Nagoya, 464-8601, Aichi, Japan.

*Corresponding author(s). E-mail(s): yamada@robo.mein.nagoya-u.ac.jp;
Contributing authors: colan@robo.mein.nagoya-u.ac.jp;
davila.ana@robo.mein.nagoya-u.ac.jp; hasegawa@mein.nagoya-u.ac.jp;

Appendix A Experimental setup

We report additional information about the experimental setup. When comparing our results to other works, we performed the evaluation methods once, which included cross-validations for JIGSAWS and the hold-out method for MISAW. In addition, we report the results of skill recognition to show the further versatility of our method in this supplementary material.

A.1 Dataset

JIGSAWS comprises three surgical tasks on the da Vinci Surgical System: 39 trials of suturing (SU), 36 trials of knot tying (KT), and 28 trials of needle passing (NP). Eight surgeons performed each task five times and were classified into three skill levels based on their robotic surgical experience: *expert* (100+ hours), *intermediate* (10-100 hours), and *novice* (<10 hours). Data corruption reduced the number of trials for each surgical task, bringing the total below 40. The surgeons controlled two patient-side

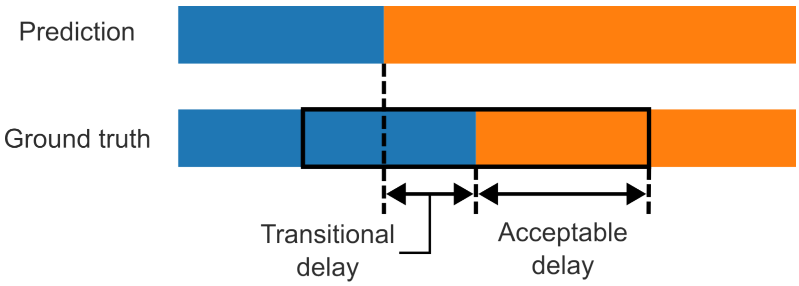

Fig. A1 Example of acceptable delay and transitional delay in the evaluation on MISAW dataset[1]

manipulators (PSMs) by the right and left master tool manipulators (MTMs) through the stereo endoscope. Demonstrations were recorded with synchronized kinematic and video data at 30 Hz. Kinematic data consists of samples of dimension 76, encompassing positions, linear velocity, rotation matrix, rotational velocity, and gripper angle velocity for left and right MTMs and two PSMs. Video data are available at 640×480 pixels from both left and right cameras. For validation, we utilize five demonstrations for SU, four for KT, and three for NP.

MISAW challenge was a one-time event for MICCAI2020, and its dataset contains 27 demonstrations of micro-surgical anastomosis on artificial blood vessels performed by three surgeons and three engineering students. We split demonstrations into 14 for training, 3 for validation, and 10 for testing. The dataset provides stereo-microscope frames (920×540).

A.2 Skill recognition

Our method utilizes unsupervised MVAE representations, the same ones used for workflow recognition, to recognize skill labels by LSTM. Skill recognition labels are denoted as $S = \{\boldsymbol{s}^d\}_{d=1}^D$, consisting of skill labels $\boldsymbol{s}_d$ for each demonstration. LSTM classifier is trained on a cross-entropy loss as a discriminative model for offline skill recognition, represented as $p(\boldsymbol{s}_d|\boldsymbol{z}_{1:T_d})$.

To validate the performance, we conducted three skill classification using LOSO cross-validation on the JIGSAWS dataset. For LSTM training, we downsampled the representations to 1 FPS and did not apply dropout, unlike workflow recognition.

A.3 Metrics

In gesture recognition on JIGSAWS, we use the following metrics outlined in [2], referencing the code in [3]. We calculate these metrics for each demonstration and compute mean and standard deviation over all demonstrations:
Accuracy for gesture recognition: This metric calculates the ratio of correctly identified gestures (denoted as N_c) to the total number of frames (N) in a demonstration. The

accuracy is presented as a percentage:

$$Acc_g = \frac{N_c \times 100}{N} \tag{A1}$$

Edit Score: The edit score is calculated as the Levanshtein distance between true (τ) and predicted (γ) sequences and scaled to [0,100] based on the maximum number of segments (G) in τ or γ:

$$Edit^* = 100 \times (1 - D(\tau, \gamma))/G \tag{A2}$$

For skill recognition, we utilized accuracy:

$$Acc_s = \frac{S_c \times 100}{S} \tag{A3}$$

Here, S_c is the number of correct skill predictions, and S is the number of demonstrations.

In the case of MISAW, we utilized balanced application-dependent accuracy (AD-Accuracy), which re-estimate accuracy using acceptable delay thresholds for a translational window (Fig. A1) [1]. AD-Accuracy is calculated based on balanced accuracy (BAC) for M classes:

$$BAC = \frac{1}{M} \sum_{i=1}^{M} \frac{TP_i}{TP_i + FN_i} \tag{A4}$$

A.4 Implementation Details

We implemented our model in PyTorch 2.0.0. This model can be trained on a GPU with a minimum of approximately 20GB of memory. TCN was fed the right camera frames resized to 224×224, and data augmentation techniques were applied, including shifts, scales, rotates, brightness, and saturation. We implemented MVAE based on [4] and computed reconstruction losses (rec loss) utilizing PyTorch's distribution framework and tuned scale parameters. We summarized the hyperparameters in Table A1, which were set based on prior experience and domain expertise. In future work, we plan to conduct a comprehensive analysis of hyperparameter and their sensitivity to investigate our model's properties and enhance its performance across multiple tasks.

A.5 Comparison targets in evaluation of workflow recognition

We provide the methods and evaluation setup of comparison targets used in the main manuscript.

A.5.1 JIGSAWS

We compared our model with state-of-the-art models which can be applicable to online inference. When multiple results were reported in their papers, we selected the best

Table A1 Hyperparameters for each module

Module	Parameter	Value	Module	Parameter	Value
TCN	Network	ResNet50	MVAE	PSMs hidden layers	{200,500}
	Projection head hidden layers	{1000, 500}		Visual hidden layer	{1000}
	Positive range	6		Distribution for rec losses	laplace
	Margin range	12		Scale for visual rec loss	0.0005
	Margin for triplet loss	0.2		Scale for PSMs rec loss	0.001
	Batch size	128		β	0.1
	Learning rate	0.0005		Learning rate	0.0001
	Weight decay	0.01		Weight decay	0.1
	Epochs	100		Batch size	256
				Min epochs	25
				Max epochs	300
LSTM	Hidden units	300			
	Number of layers	1			
	Learning rate	0.001			
	Weight decay	0.05			
	Batch size	3			
	Dropout prob(workflow)	0.5			
	Epochs	70			

outcome under conditions that did not use future data to align the conditions as closely as possible. Our model was evaluated at 5 FPS and 30 FPS on both LOUO and LOSO cross-validations and predicted labels only from current and past data. It is important to acknowledge that performance differences observed in the comparisons may stem from their varying settings, such as the use of future data, frame rates (FPS), pre-processing and post-processing techniques, and different evaluation methods. In practical implementation, recognition systems must be carefully designed to balance recognition frequency, accuracy, and segmental coherence, all of which can be impacted by such factors. The summary of comparison targets is as follows. Please refer to the original papers for more details.

Forward LSTM[5] was trained using positions, velocities, and gripper angles from PSMs at 5 FPS, run online, and evaluated on LOUO cross-validation.

Skip-Chain Conditional Random Field (SC-CRF) [6, 7] utilizes data from a distant previous state instead of the neighboring one, enabling it to capture gesture transitions over a longer range than a typical Linear Chain Conditional Random Field. In the model, potential functions rely only on the current and previous state. They predict the sequences using a modified version of the Viterbi algorithm and applying a median filter. We employed the results using all PSMs at 30 FPS on LOUO cross-validation for comparison.

3D-CNN [8] utilizes a 3D convolutional neural network to extract spatiotemporal information from the video. We employed the result of their model, which initialized with pre-trained weights on Kinetics [9], did not incorporate future data, and evaluated at 5 FPS on LOUO cross-validation.

Fusion-KV [10] first predicts the workflow labels for each modality data. Visual data are processed by a combination of CNN pre-trained on ImageNet and a Temporal Convolutional Network. Additionally, a forward LSTM and a Temporal Convolutional

Network process normalized kinematic data, which includes positions, velocities, gripper angles, and Euler angles from PSMs. These predictions are then combined using a weighted voting method. The evaluation was carried out using LOUO cross-validation. It's worth noting that although this model is applicable to online inference, the results on the JIGSAWS dataset were derived from non-causal convolutional layers, which uses future data.

MRG-NET [11] first creates latent representation from video and kinematic data with Temporal Convolutional Networks and LSTM units. Then, these features from each modality are integrated through a relational graph convolutional network. For kinematic input, they convert the rotation matrix into Eular angles, then normalized all data from PSMs to zero mean and unit variance. Their evaluation on the JIGSAWS dataset, carried out using LOUO cross-validation, did not specify the FPS value and clarify if causal convolutions were employed for their stated "online approach".

MA-TCN [12] processes separately visual and kinematic data by Temporal Convolutional Networks (TCN) and then integrates them while weighting dynamically in time through multimodal attention. They utilized smoothed and normalized positions, velocities, and gripper angles from PSMs. The model was evaluated at 5 FPS on LOUO cross-validation with rectification of the 12 annotation errors. Our model was compared with the model in the casual setting, which only relies on past and current data.

Motion2vec [13] uses contrastive learning with partially labeled data to learn a latent space that reflects manual and generated gesture labels and predicted segment labels by multiple classifiers. They treated a total of 78 videos from two available streams of stereo cameras as a complete dataset and downsample them at 3 frames per second with an average duration of 3 minuted per video. The evaluation was based on the average accuracy calculated over four iterations of the LOSO test set, which were created by random choice of four demonstrations from each surgeon for the training set. We employed the condition with the highest performance except for the bi-directional LSTM, which uses future data. The condition used a combination of labeled triplet and sv-TCN loss for representation learning on a CNN, which initialized ImageNet pre-trained weights and the k-nearest neighbor for a classifier. This combination can operate without future data.

Cross-modal [14] first trains an encoder-decoder network by predicting kinematic data from optical flows and then freezes the weights and truncates the decoder. The representation from the encoder is fed into the XGBoost classifier to recognize gestures. To align the condition, we used results where the training and test data were identical. They conducted gesture classification, a segmental classification that predicts a gesture for each segment based on the assumption that the boundaries of each gesture are known. Gesture recognition performed by all the others is frame-wise classification and significantly more challenging, as it requires the simultaneous determination of gesture boundaries and labels, i.e., a finer and more dynamic understanding of the scene [6]. Regardless, the comparison remains valid as both tasks evaluate the model's performance by recognizing the corresponding gesture for the given data. In addition, they employed balanced leave-one-trial-out (LOTO) cross-validation, which takes one trial for the test set while balancing the gesture classes.

On the other hand, we employed leave-one-supertrial-out (LOSO) cross-validation, extending LOTO cross-validation to a broader array of demonstrations, taking one trial from each surgeon. They both assess model performance to new demonstrations; while the comparison is valid, LOSO is more difficult due to the need for generalizability across all surgeons. In summary, this comparison, though not a direct equivalence due to the outlined differences, provides valuable insights. Despite facing more challenging conditions, our method demonstrated significant performance improvements, highlighting the effectiveness of our multimodal representation learning. These results underscore the superiority of our approach in complex scenarios.

A.5.2 MISAW

For comparison in phase and step recognition, our main manuscript used the MISAW challenge report models, including six uni-task and multi-task learning models. The performances of multi-task models depend on their ability to capture relationships between multiple tasks, potentially leading to synergistic benefits. However, if they cannot effectively capture the relationships, the tasks' complexity increases. To assess each granularity's performance directly, we compared our model against the following four uni-task models except for two models with only multi-task learning.

UniandesBCV extracted the features of video frames using ResNet and fed it into a SlowFast model pre-trained on Kinetics. Wr0112358 also employed a vision-based model, a DenseNet121 CNN, with data augmentation and regularization. MedAir utilized MRG-Net[11] for workflow recognition based on both video and kinematic data. They correct prediction based on a median filter and a post-processing strategy (PKI). IMPACT also used video and kinematic data. Video data were rescaled and normalized, and kinematic data were normalized to have zero mean and unit variance. In addition, data were downsampled to 5 FPS. A pre-trained VGG19 on ImageNet processed video data and an adapted ResNet network processed kinematic data, and then the output from each modality network was concatenated to predict workflow labels. For more details about these models, you can refer to [1].

Appendix B Additional Results and Discussion

B.1 Visualization of a latent representation

We show comprehensive UMAP visualizations of the representations from TCN and MVAE on the JIGSAWS dataset in Fig. B2, B3, B4, B5, B6, B7, B8. Particularly in tasks involving Suturing (SU) and Knot Tying (KT), TCN can solidify each gesture, implying that TCN effectively breaks down the surgical process into gesture-level components by comprehending the complex video sequences. However, we observe a different pattern when we examine the Needle Passing (NP) task. TCN demonstrates proficiency in understanding the sequential properties of NP but does not effectively separate the distribution by individual gestures. This outcome suggests that in NP, there might be a limited connection between the video flow and the gestures, making it challenging to deconstruct the representation into gesture-level components.

Furthermore, we visualized the output of the projection head represented by $\boldsymbol{b}$ in Fig. B5. These visualizations demonstrate that $\boldsymbol{b}$ focuses on only the temporal dimension, which strongly compresses the latent space. A comparison between Fig. B2 and Fig. B5 reveals the impact of the projection head incorporation, which mitigates the intense compression while preserving the sequential property.

MVAE forms clusters of gesture labels, with some areas showing the gathering of temporal neighbors. In addition, it effectively forms distinct clusters that align with different skill levels, as shown in Fig. B6b, B7b, B8b. This highlights MVAE's ability to differentiate skill levels by incorporating kinematic data. Notably, the MVAE visualization separates *novice* and *expert*, with *intermediate* positioned as one of them. The *intermediate* definition in JIGSAWS represents a transition from *novice* to *expert*. This distribution may result from two categories of *intermediate*: some closer to *novice* and others closer to *expert*. This pattern is consistent with distributions observed in previous representation learning approach [14] and reinforces previous findings of lower recognition accuracy among *intermediate* in skill assessment studies [15]. These visualizations demonstrate MVAE's ability to distinguish skill levels and gestures while holding temporal context.

In Fig. B10, B11, we conducted four visualizations of these representations using the MISAW dataset: normalized frame indexes (video sequence), phase labels, step labels, and demonstration indexes. TCN captures some local information, such as phase and step, but struggles to capture the global video sequence. This shows the difficulty of time contrastive learning in tasks with multi-stage complex processes.

Conversely, the introduction of MVAE leads to significant enhancements in the quality of representation. MVAE creates clusters based on the characteristics of each demonstration, as shown in the visualization of the video indexes (Fig. B11). This capability allows MVAE to effectively express the sequential features, phases, and steps on each cluster as shown in Fig. B10d, Fig. B10e, and Fig. B10f. By combining kinematic data, organizing information, and distinguishing features for each demonstration, MVAE facilitates a hierarchical understanding of the workflow.

These findings underscore the synergistic benefits of combining TCN and MVAE and the high generalization performance of MVAE.

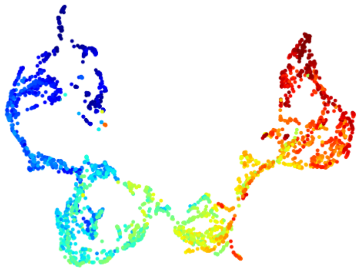

(a) SU sequence

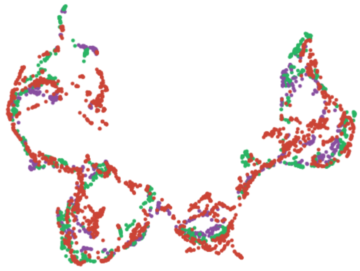

(b) SU skill

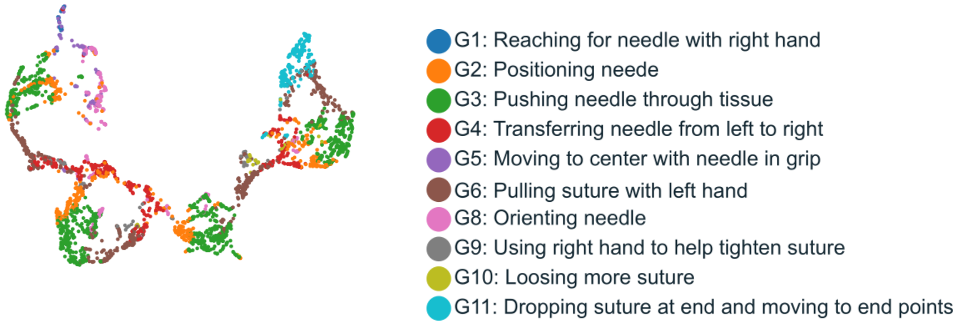

(c) SU gesture

Fig. B2 TCN visualization (visual feature $\boldsymbol{v}$) on JIGSAWS Suturing: "sequence" is colored by frame indexes normalized from 0.0 (blue) to 1.0 (red) on each demonstration, "gesture" is colored by gesture labels, and "skill" is colored by *novice* (red), *intermediate* (purple), *expert* (green)

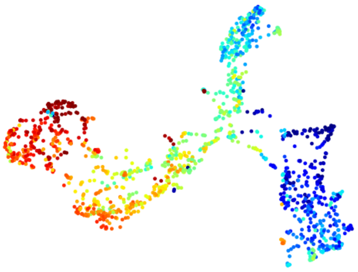

(a) KT sequence

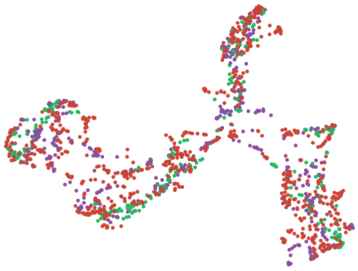

(b) KT skill

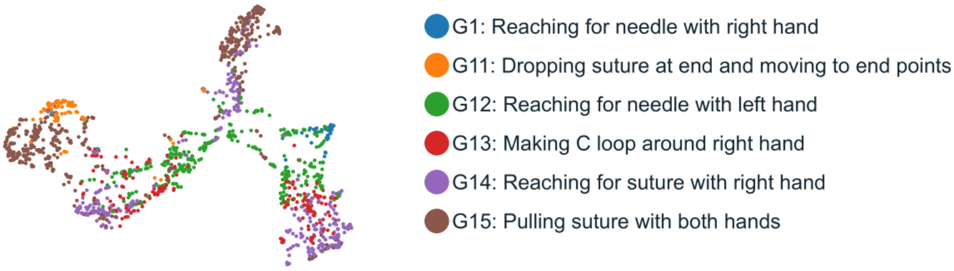

(c) KT gesture

Fig. B3 TCN visualization (visual feature $\boldsymbol{v}$) on JIGSAWS Knot Tying: "sequence" is colored by frame indexes normalized from 0.0 (blue) to 1.0 (red) on each demonstration, "gesture" is colored by gesture labels, and "skill" is colored by *novice* (red), *intermediate* (purple), *expert* (green)

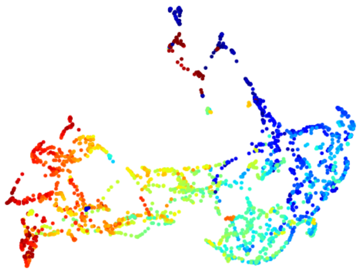

(a) NP sequence

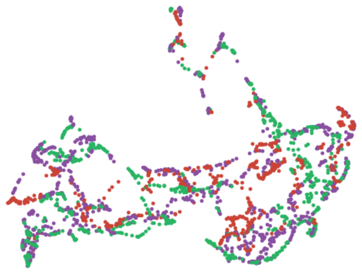

(b) NP skill

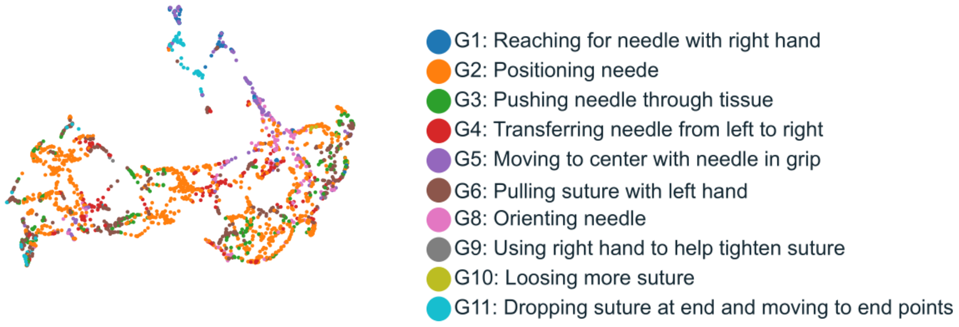

(c) NP gesture

Fig. B4 TCN visualization (visual feature $\boldsymbol{v}$) on JIGSAWS Needle Passing: "sequence" is colored by frame indexes normalized from 0.0 (blue) to 1.0 (red) on each demonstration, "gesture" is colored by gesture labels, and "skill" is colored by *novice* (red), *intermediate* (purple), *expert* (green)

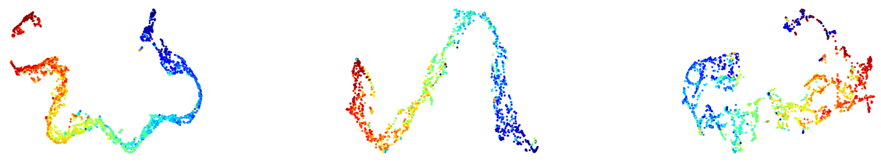

(a) SU sequence (b) KT sequence (c) NP sequence

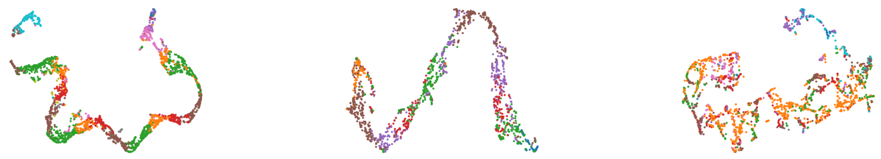

(d) SU gesture (e) KT gesture (f) NP gesture

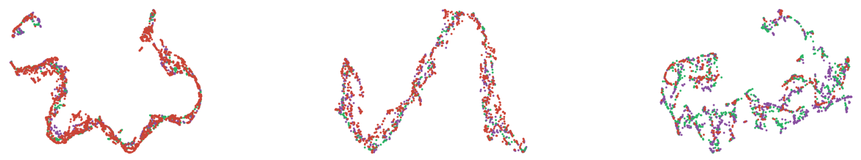

(g) SU skill (h) KT skill (i) NP skill

Fig. B5 TCN visualization (projected feature $\boldsymbol{b}$) on JIGSAWS: "sequence" is colored by frame indexes normalized from 0.0 (blue) to 1.0 (red) on each demonstration, "gesture" is colored by gesture labels, and "skill" is colored by *novice* (red), *intermediate* (purple), *expert* (green)

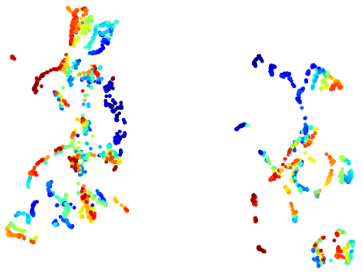

(a) SU sequence

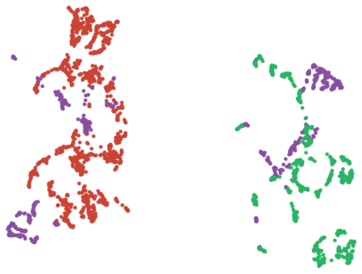

(b) SU skill

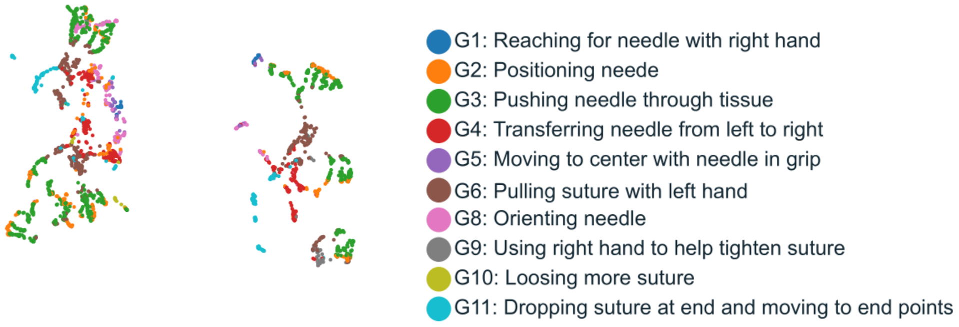

(c) SU gesture

Fig. B6 MVAE visualization on JIGSAWS Suturing: "sequence" is colored by frame indexes normalized from 0.0 (blue) to 1.0 (red) on each demonstration, "gesture" is colored by gesture labels, and "skill" is colored by *novice* (red), *intermediate* (purple), *expert* (green)

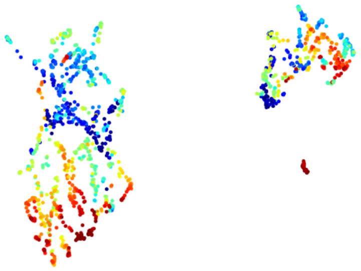

(a) KT sequence

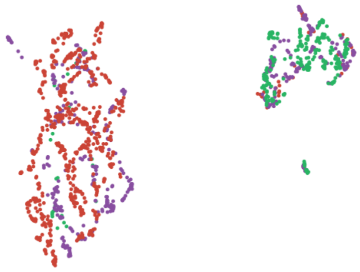

(b) KT skill

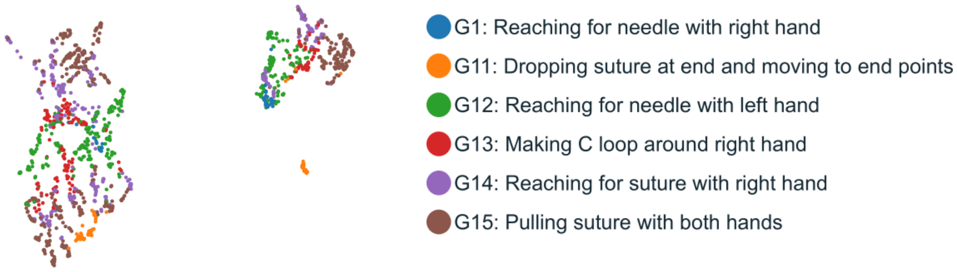

(c) KT gesture

Fig. B7 MVAE visualization on JIGSAWS Knot Tying: ”sequence” is colored by frame indexes normalized from 0.0 (blue) to 1.0 (red) on each demonstration, ”gesture” is colored by gesture labels, and ”skill” is colored by *novice* (red), *intermediate* (purple), *expert* (green)

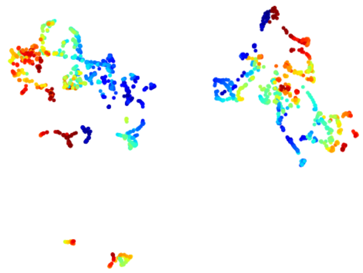

(a) NP sequence

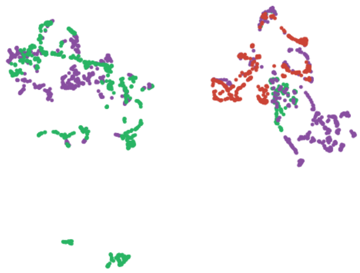

(b) NP skill

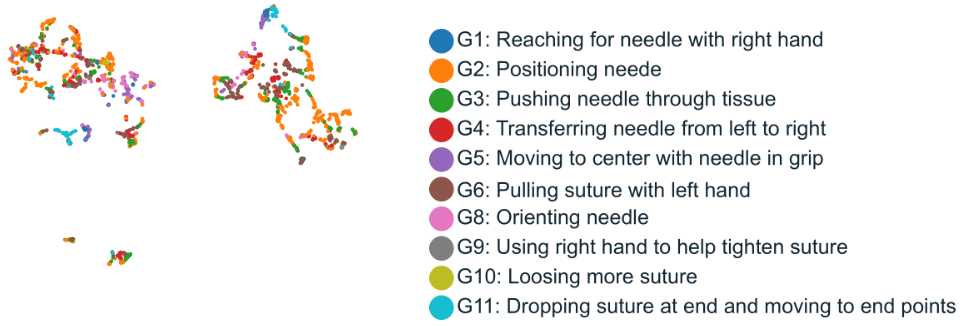

(c) NP gesture

Fig. B8 MVAE visualization on JIGSAWS Needle Passing: ”sequence” is colored by frame indexes normalized from 0.0 (blue) to 1.0 (red) on each demonstration, ”gesture” is colored by gesture labels, and ”skill” is colored by *novice* (red), *intermediate* (purple), *expert* (green)

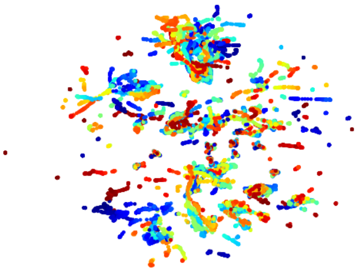

(a) SU sequence

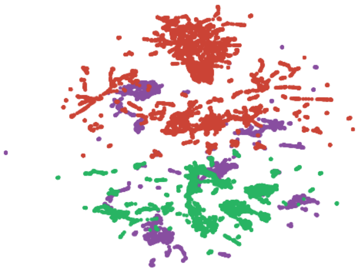

(b) SU skill

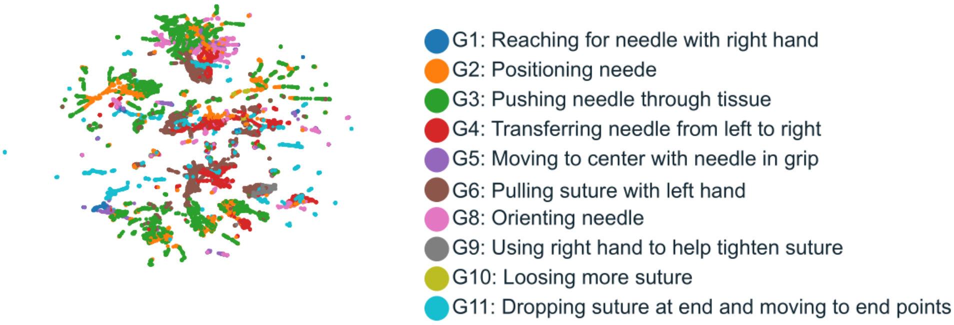

(c) SU gesture

Fig. B9 Visualization of robot arms kinematic data applied standardization on JIGSAWS Suturing: "sequence" is colored by frame indexes normalized from 0.0 (blue) to 1.0 (red) on each demonstration, "gesture" is colored by gesture labels, and "skill" is colored by *novice* (red), *intermediate* (purple), *expert* (green)

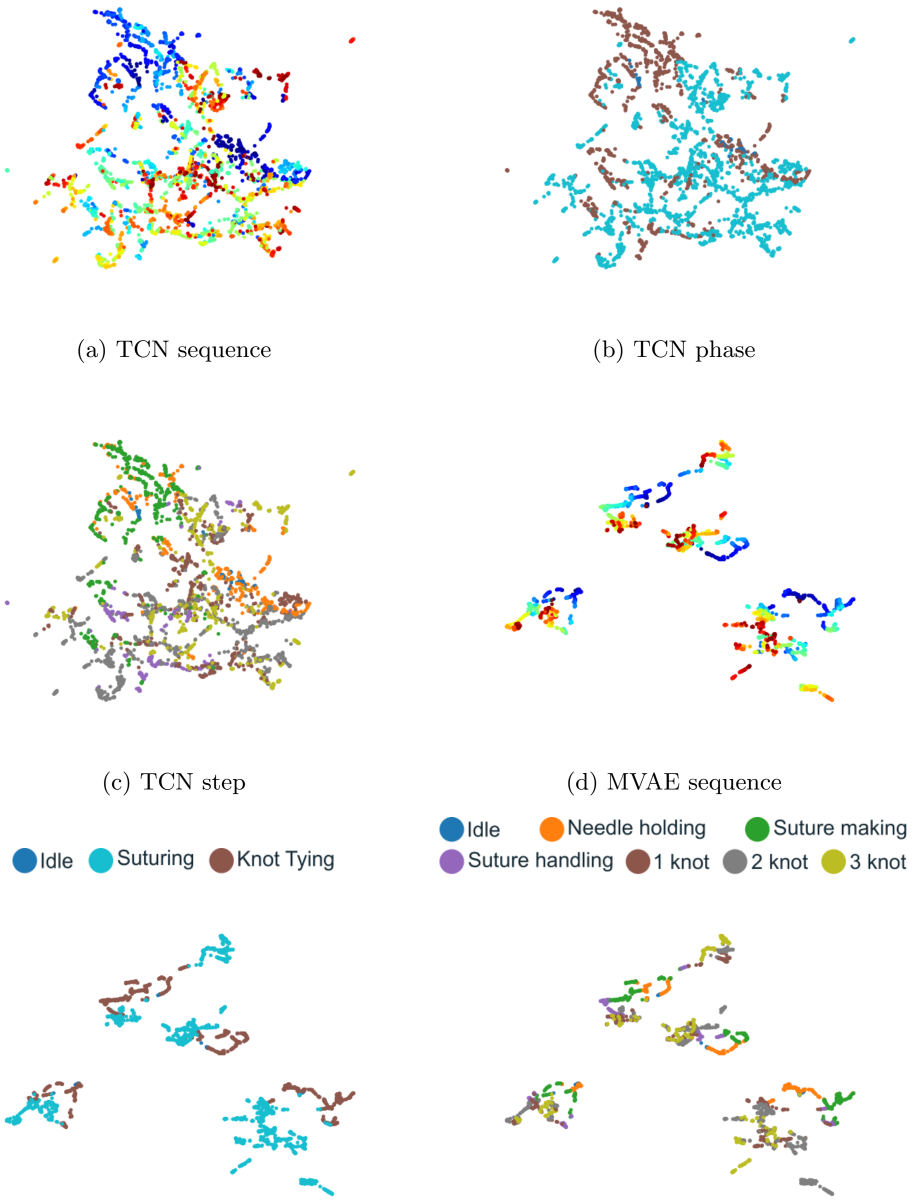

(a) TCN sequence (b) TCN phase

(c) TCN step (d) MVAE sequence

(e) MVAE phase (f) MVAE step

Fig. B10 MISAW visualization: visual feature $\boldsymbol{v}$ is utilized in TCN visualization, ”sequence” is colored by frame indexes normalized from 0.0 (blue) to 1.0 (red) on each demonstration, ”phase” and ”step are colored by each label

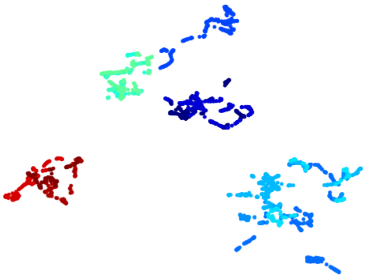

Fig. B11 MVAE visualization colored by demonstration indexes on MISAW

Table B2 Gesture recognition performance on JIGSAW SU, KT, and NP

		SU		KT		NP	
Valid	Avg. Time	Acc	Edit*	Acc	Edit*	Acc	Edit*
LOUO	15.8h (4.7)	83.3(8.3)	61.8(17.3)	75.5(14.6)	51.7(18.2)	64.3(14.0)	37.2(12.7)
LOSO	9.5h (2.1)	87.9(6.6)	71.5(15.9)	77.2(13.8)	53.8(20.3)	71.5(17.3)	47.7(19.4)

B.2 Results of gesture recognition

We present the results for JIGSAWS Suturing, Knot Tying, and Needle Passing in Table B2. We observed a significant performance drop in gesture recognition in NP compared with SU and KT. This drop in performance can be attributed to TCN's challenges in comprehending gestures in NP, as previously discussed. These findings indicate that our model's representations might not always align perfectly with human intent, partly due to our reliance on unlabeled data. This insufficient representation learning could also contribute to the failure to recognize the gestures G9 and G10. To cope with these issues, future work should explore efficient ways to incorporate annotation information, such as fine-tuning and active learning strategies.

As detailed in Section 5.3 of the main manuscript and as observed in the UMAP visualization, our representation learning could be influenced by variability in small datasets, including variations in skill level and individual demonstration characteristics. Insufficient data hinders generalization and promotes overfitting, resulting in difficulty in learning robust representations. Differences in data volume across JIGSAWS tasks are one of the contributing factors to the lower and less stable performance on the KT and NP datasets in the challenging cross-validations of evaluating generalization performance for an unseen surgeon or unseen demonstrations from all surgeons.

Table B3 Skill recognition performance on JIGSAW SU, KT, and NP using LOSO cross-validation

Model	Type	Avg. Time	Accuracy SU	KT	NP
3D-CNN[15]	Sup	–	**100**	**95.1**	**100**
Cross-modal[14][1]	Semi	–	67	81	63
Ours	Semi	1.5h (0.4)[2]	87.2	86.1	96.4

[1]Evaluated on leave-one-trial-out.

[2]We trained LSTM only.

Table B4 The effect of FPS in gesture recognition performance

FPS	Accuracy	Edit
1	82.1(9.0)	85.8(10.2)
5	82.4(9.2)	76.7(15.2)
10	83.2(8.3)	74.3(16.4)
20	83.7(8.4)	63.6(19.0)
30	83.3(8.3)	61.8(17.3)

Evaluated on LOUO cross-validation on JIGSAWS SU

B.3 Results of skill recognition

We assess our representation's capacity for skill recognition using the same representation used for gesture recognition. We compared our results with a fully supervised model specifically trained for skill recognition using 3D-CNN [15] and a model that conducted similar experiments recognizing both gesture and skill from one representation [14], as presented in Table B3. Our model achieved an approximately 10% lower accuracy than the supervised skill recognition model. However, compared with [14], our model exhibited a remarkable performance improvement of nearly 20% for SU and NP. As shown in Fig. B12, most of the prediction errors are associated with intermediate, aligning with the observations from the visualization of latent representation. Skill labels in this study were determined based on participants' hours of experience in robotic surgery. However, these labels did not account for the performance quality in each demonstration. The variability at the instance level within the intermediate could significantly influence the performance.

In addition to multi-granularity recognition, our model demonstrates the ability to discern skill levels, showcasing the further versatility of our representation learning. Looking ahead, multi-task learning for both skill and workflow recognition appears promising for enhancing performance in both cases. As shown in the visualization, our representation forms clusters based on skill levels, suggesting a potential synergistic effect when the model is trained while considering the relationship between skills and workflow. In addition, this capability allows for advanced surgical training platforms. These could offer real-time instructions tailored to current behavior and assess skill levels upon completion.

Furthermore, this model can recognize a surgeon's skill online by changing the LSTM classifier from a current mechanism that holds the cell state in the whole sequence to one that operates within a constant window similar to [16]. This shift could expand an application range, such as an immediate surgical quality assessment and the dynamic adjustment of support level based on the quality of surgeons' behavior, thereby providing more adaptable and responsive assistance.

Table B5 Video duration in MISAW

Participants	Duration[min]
Students	4.0(2.0)
Experts	2.5(0.7)
**All**	**3.4(1.8)**

Measured by OpenCV

Table B6 Phase and Step recognition performance on MISAW

Task	Time	AD-Accuracy	AD-F1
Phase	1.2h[1]	84.0(15.0)	82.2(23.4)
Step	2.5h	56.8(9.3)	49.5(13.3)

[1]We trained LSTM only.

B.4 Results on the MISAW dataset

As mentioned in the main text, lower performance than supervised models could be due to high variability in a small dataset. In the MISAW dataset, surgeons' videos average 2.5 minutes in length, while students' average 4.0 minutes, and the dataset exhibits a standard deviation of 1.8 minutes for the total lengths (Table B5). High variation in video duration might affect the LSTM we employed. The current LSTM's mechanism, designed to hold the cell state throughout the entire video, may encounter difficulties with state updates due to the difference in video length, especially as the video progresses. This is illustrated by the noticeable decline in performance after *Suture making*, as shown in the confusion matrix (Fig. B15f). To better accommodate the properties of specific surgical tasks, exploring alternatives like a temporal convolutional network [17] or LSTM with sliding window, which processes data in fixing time segments, could be beneficial over the continuous sequence approach of the current LSTM model.

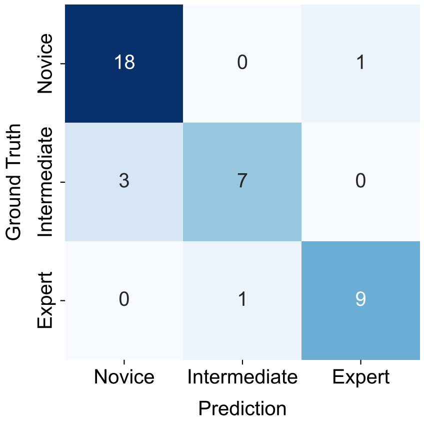

(a) SU

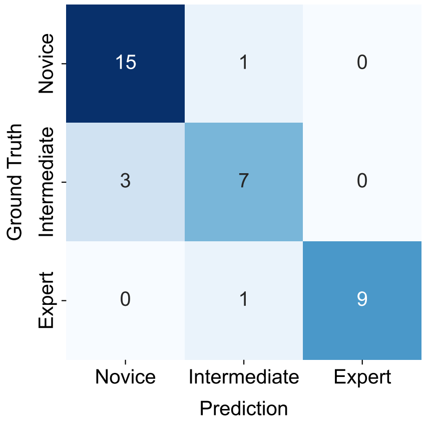

(b) KT

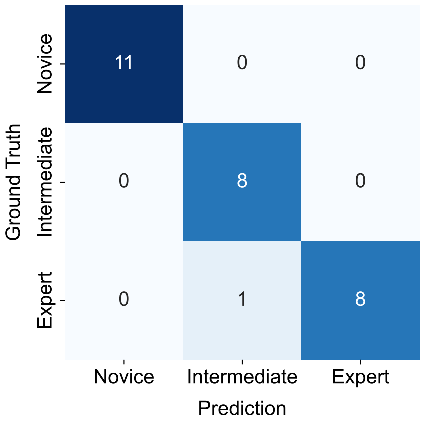

(c) NP

Fig. B12 Confusion matrix of skill recognition on JIGSAWS using LOSO cross-validation

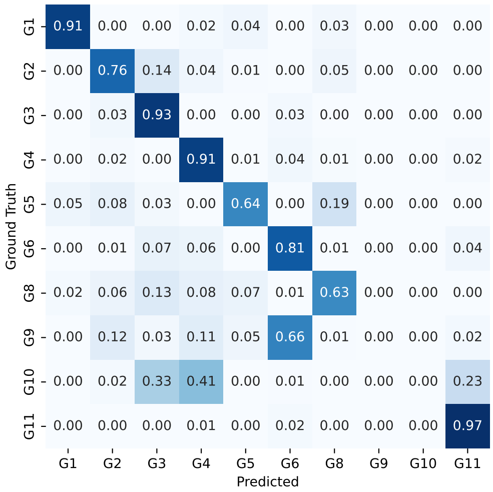

(a) JIGSAWS SU on LOUO

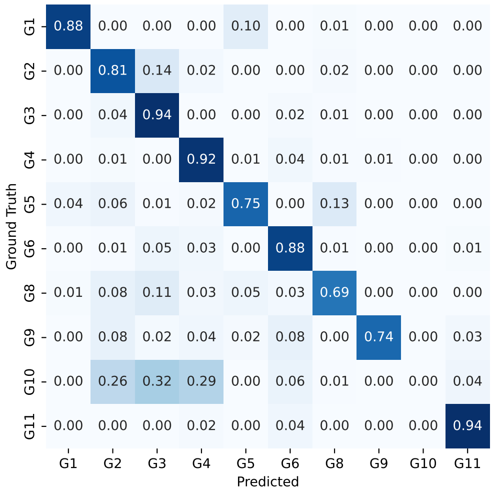

(b) JIGSAWS SU on LOSO

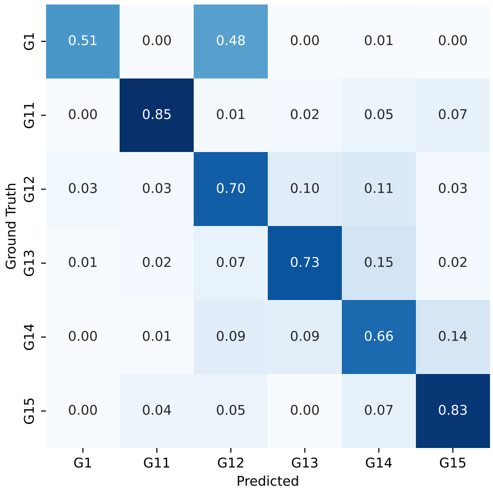

(c) JIGSAWS KT on LOUO

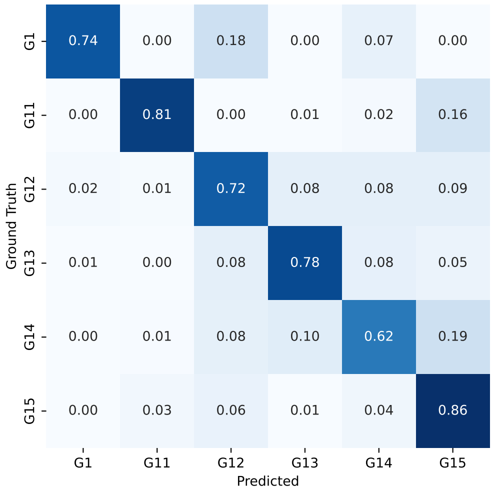

(d) JIGSAWS KT on LOSO

Fig. B13 Confusion matrix of gesture recognition on JIGSAWS SU and KT

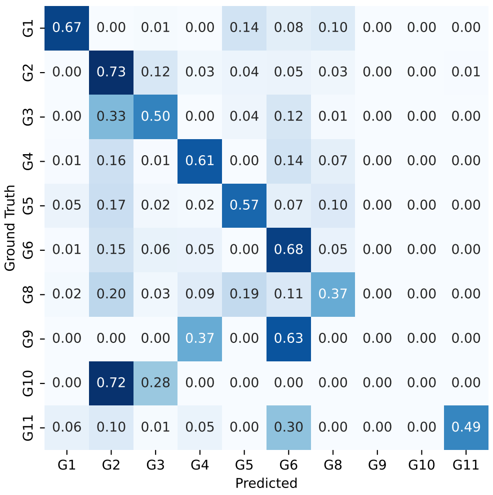

(a) JIGSAWS NP on LOUO

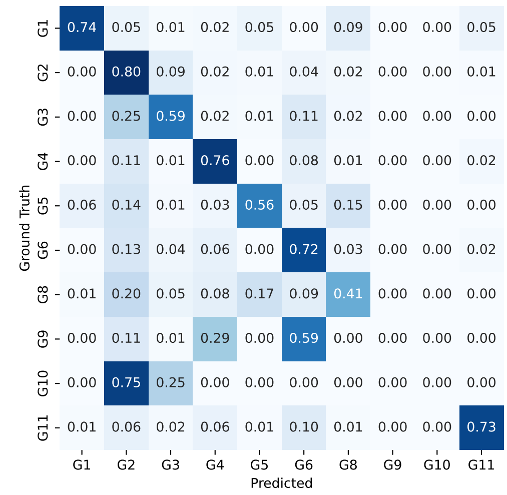

(b) JIGSAWS NP on LOSO

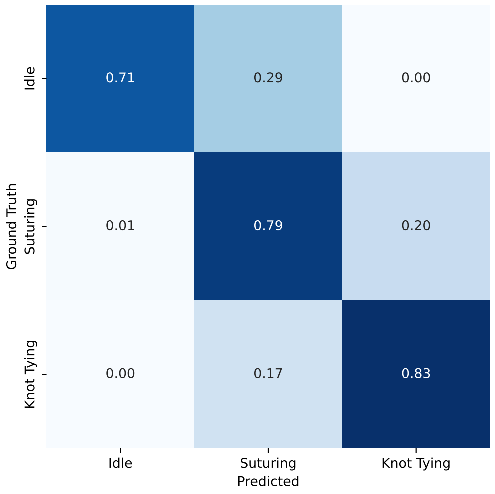

(c) MISAW phase

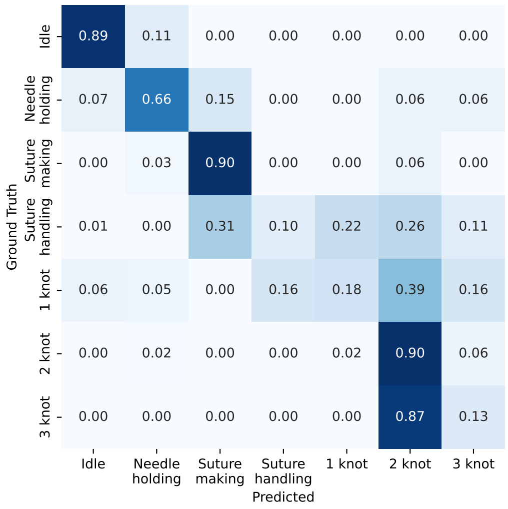

(d) MISAW step

Fig. B14 Confusion matrix of gesture recognition on JIGSAW NP, phase and step recognition on MISAW

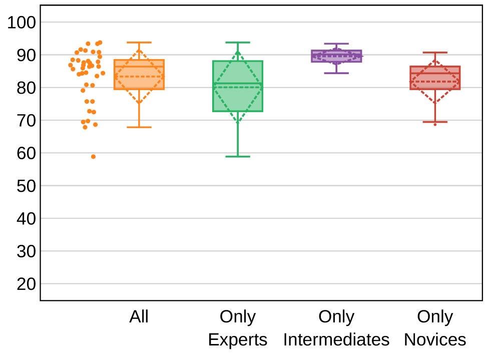

(a) JIGSAWS SU on LOUO

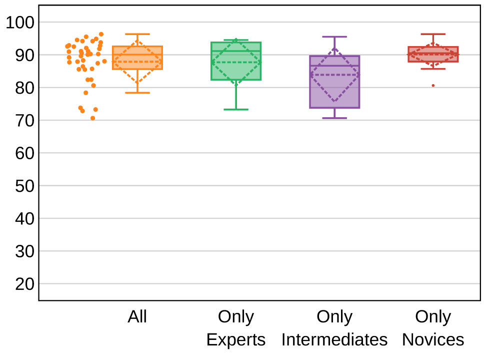

(b) JIGSAWS SU on LOSO

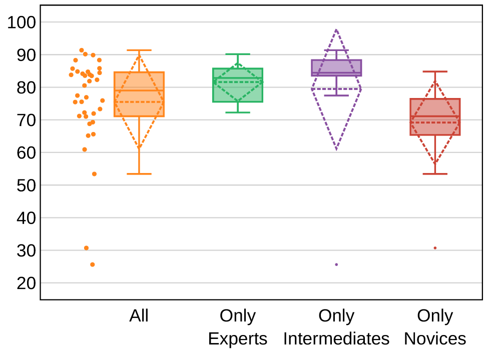

(c) JIGSAWS KT LOUO

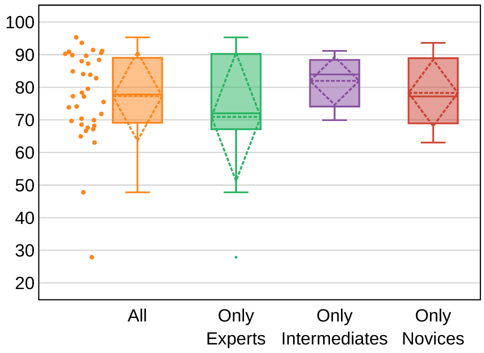

(d) JIGSAWS KT LOSO

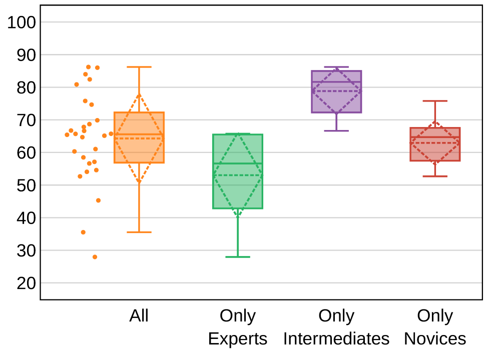

(e) JIGSAWS NP on LOUO

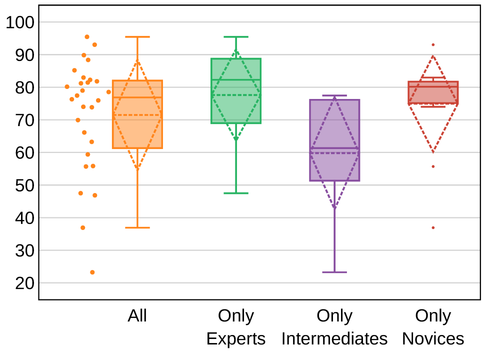

(f) JIGSAWS NP on LOSO

Fig. B15 Test Accuracy on JIGSAWS: dash lines represent mean and standard deveation

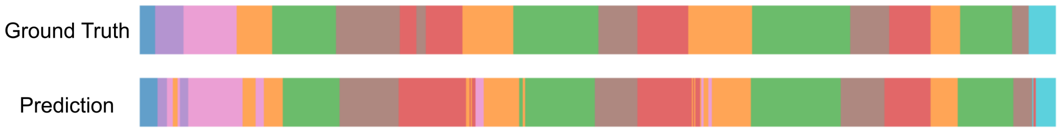

(a) Median. Accuracy (86.4%) on JIGSAWS SU

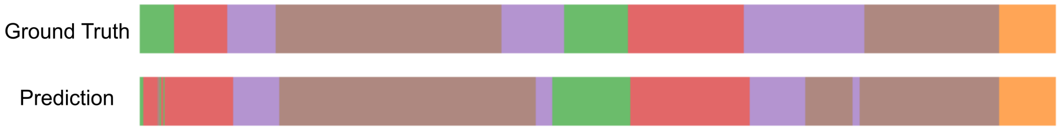

(b) Closest below median. Accuracy (77.5%) on JIGSAWS KT

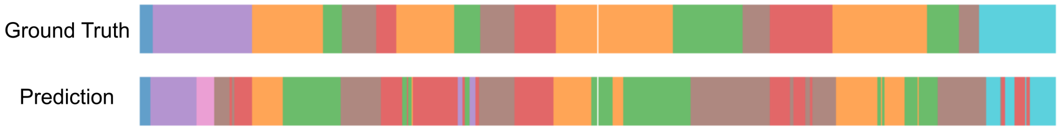

(c) Closest below median. Accuracy (65.4%) on JIGSAWS NP

Fig. B16 Color-coded ribbon visualization of median (or closest below median) on JIGSAW SU using LOUO cross-validation

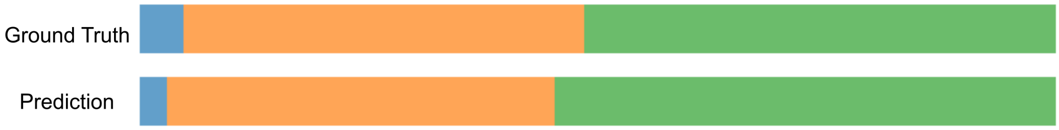

(a) Closest below median. AD-accuracy (84.3%) on MISAW phase recognition

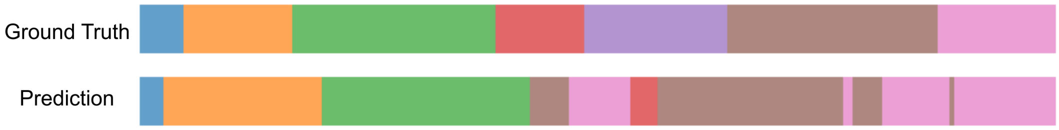

(b) Closest below median. AD-accuracy (57.6%) on MISAW step recognition

Fig. B17 Color-coded ribbon visualization of closest below median on MISAW

References

[1] Huaulmé, A., Sarikaya, D., Le Mut, K., Despinoy, F., Long, Y., Dou, Q., Chng, C.-B., Lin, W., Kondo, S., Bravo-Sánchez, L., *et al.*: Micro-surgical anastomose workflow recognition challenge report. Computer Methods and Programs in Biomedicine **212**, 106452 (2021)

[2] Amsterdam, B., Clarkson, M.J., Stoyanov, D.: Gesture recognition in robotic surgery: a review. IEEE Transactions on Biomedical Engineering **68**(6) (2021)

[3] Lea, C., Flynn, M.D., Vidal, R., Reiter, A., Hager, G.D.: Temporal convolutional networks for action segmentation and detection. In: Proceedings of the IEEE Conference on Computer Vision and Pattern Recognition, pp. 156–165 (2017)

[4] Sutter, T.M., Daunhawer, I., Vogt, J.E.: Generalized multimodal ELBO. In: International Conference on Learning Representations (2021)

[5] DiPietro, R., Lea, C., Malpani, A., Ahmidi, N., Vedula, S.S., Lee, G.I., Lee, M.R., Hager, G.D.: Recognizing surgical activities with recurrent neural networks. In: Medical Image Computing and Computer-Assisted Intervention–MICCAI 2016: 19th International Conference, Athens, Greece, October 17-21, 2016, Proceedings, Part I 19, pp. 551–558 (2016). Springer

[6] Ahmidi, N., Tao, L., Sefati, S., Gao, Y., Lea, C., Haro, B.B., Zappella, L., Khudanpur, S., Vidal, R., Hager, G.D.: A dataset and benchmarks for segmentation and recognition of gestures in robotic surgery. IEEE Transactions on Biomedical Engineering **64**(9), 2025–2041 (2017)

[7] Lea, C., Hager, G.D., Vidal, R.: An improved model for segmentation and recognition of fine-grained activities with application to surgical training tasks. In: 2015 IEEE Winter Conference on Applications of Computer Vision, pp. 1123–1129 (2015). https://doi.org/10.1109/WACV.2015.154

[8] Funke, I., Bodenstedt, S., Oehme, F., Bechtolsheim, F., Weitz, J., Speidel, S.: Using 3d convolutional neural networks to learn spatiotemporal features for automatic surgical gesture recognition in video. In: International Conference on Medical Image Computing and Computer-assisted Intervention, pp. 467–475 (2019). Springer

[9] Carreira, J., Zisserman, A.: Quo vadis, action recognition? a new model and the kinetics dataset. In: Proceedings of the IEEE Conference on Computer Vision and Pattern Recognition, pp. 6299–6308 (2017)

[10] Qin, Y., Pedram, S.A., Feyzabadi, S., Allan, M., McLeod, A.J., Burdick, J.W., Azizian, M.: Temporal segmentation of surgical sub-tasks through deep learning with multiple data sources. In: Proc. of IEEE Intl Conf. on Robotics and Automation (ICRA), pp. 371–377 (2020). IEEE

[11] Long, Y., Wu, J.Y., Lu, B., Jin, Y., Unberath, M., Liu, Y.-H., Heng, P.A., Dou, Q.: Relational graph learning on visual and kinematics embeddings for accurate gesture recognition in robotic surgery. In: Proc. of IEEE Intl Conf. on Robotics and Automation (ICRA), pp. 13346–13353 (2021). IEEE

[12] Van Amsterdam, B., Funke, I., Edwards, E., Speidel, S., Collins, J., Sridhar, A., Kelly, J., Clarkson, M.J., Stoyanov, D.: Gesture recognition in robotic surgery with multimodal attention. IEEE Transactions on Medical Imaging **41**(7), 1677–1687 (2022)

[13] Tanwani, A.K., Sermanet, P., Yan, A., Anand, R., Phielipp, M., Goldberg, K.: Motion2vec: Semi-supervised representation learning from surgical videos. In: Proc. of IEEE Intl Conf. on Robotics and Automation (ICRA), pp. 1–8 (2020)

[14] Wu, J.Y., Tamhane, A., Kazanzides, P., Unberath, M.: Cross-modal self-supervised representation learning for gesture and skill recognition in robotic surgery. International Journal of Computer Assisted Radiology and Surgery **16**, 779–787 (2021)

[15] Funke, I., Mees, S.T., Weitz, J., Speidel, S.: Video-based surgical skill assessment using 3d convolutional neural networks. International journal of computer assisted radiology and surgery **14**, 1217–1225 (2019)

[16] Wang, Z., Majewicz Fey, A.: Deep learning with convolutional neural network for objective skill evaluation in robot-assisted surgery. International journal of computer assisted radiology and surgery **13**, 1959–1970 (2018)

[17] Czempiel, T., Paschali, M., Keicher, M., Simson, W., Feussner, H., Kim, S.T., Navab, N.: Tecno: Surgical phase recognition with multi-stage temporal convolutional networks. In: International Conference on Medical Image Computing and Computer-assisted Intervention, vol. 12263, pp. 343–352 (2020)
